# Bulk elastic moduli and solute potentials in leaves of freshwater, coastal and marine hydrophytes. Are marine plants more rigid?

**DOI:** 10.1093/aobpla/plu014

**Published:** 2014-03-28

**Authors:** Brant W. Touchette, Sarah E. Marcus, Emily C. Adams

**Affiliations:** 1 Department of Environmental Studies, Elon University, Elon, NC 27244, USA; 2 Present address: Department of Biology, Old Dominion University, Norfolk, VA 23529, USA

**Keywords:** Bulk elastic modulus, halophytes, hydrophytes, salinity, solute potential, symplastic water content.

## Abstract

Bulk modulus of elasticity (*ɛ*), depicting the flexibility of plant tissues, is recognized as an important component in maintaining internal water balance. Elevated *ɛ* and comparatively low osmotic potential (*Ψ_π_*) may work in concert to effectively maintain vital cellular water content. This concept, termed the ‘cell water conservation hypothesis’, may foster tolerance for lower soil-water potentials in plants while minimizing cell dehydration and shrinkage. Therefore, the accumulation of solutes in marine plants, causing decreases in *Ψ_π_*, play an important role in plant–water relations and likely works with higher *ɛ* to achieve favourable cell volumes. While it is generally held that plants residing in marine systems have higher leaf tissue *ɛ*, to our knowledge no study has specifically addressed this notion in aquatic and wetland plants residing in marine and freshwater systems. Therefore, we compared *ɛ* and *Ψ_π_* in leaf tissues of 38 freshwater, coastal and marine plant species using data collected in our laboratory, with additional values from the literature. Overall, 8 of the 10 highest *ɛ* values were observed in marine plants, and 20 of the lowest 25 *ɛ* values were recorded in freshwater plants. As expected, marine plants often had lower *Ψ_π_*, wherein the majority of marine plants were below −1.0 MPa and the majority of freshwater plants were above −1.0 MPa. While there were no differences among habitat type and symplastic water content (*θ*_sym_), we did observe higher *θ*_sym_ in shrubs when compared with graminoids, and believe that the comparatively low *θ*_sym_ observed in aquatic grasses may be attributed to their tendency to develop aerenchyma that hold apoplastic water. These results, with few exceptions, support the premise that leaf tissues of plants acclimated to marine environments tend to have higher *ɛ* and lower *Ψ_π_*, and agree with the general tenets of the cell water conservation hypothesis.

## Abstract (precis)

The flexibility of plant cell walls is characterized by bulk modulus of elasticity (*ϵ*); which is an important component of how plants maintain adequate water continent. For example, plants with rigid tissues (high *ϵ*) that accumulate solutes may better tolerate drought or saline soils. This concept is termed the ‘cell water conservation hypothesis.’ While it is generally held that marine plants have higher *ϵ*, no study has considered that notion across a number of species residing in marine and coastal habitats. The finding from this study show that aquatic marine plants do maintain rigid tissues with lower osmotic potentials (relative to freshwater plants), and support the tenets of the cell water conservation hypothesis

## Introduction

The flexibility of plant tissues, as characterized by bulk modulus of elasticity (*ɛ*), is an important component of plant–water relations (Steudle *et al.* 1977; Joly and Zaerr 1987). Plants with relatively flexible cell walls (low *ɛ*) can maintain adequate turgor during periods of tissue-water decline (Clifford *et al.* 1998). In contrast, rigid tissues restrict appreciable changes in cell size, and it has been suggested that limiting cellular water flux could help maintain intermolecular distances within cell fluids supporting optimal metabolic function (e.g. maintaining appropriate cellular pH or preventing ‘salting out’ of important metabolites; Jones 1973; Jones and Corlett 1992; Clifford *et al.* 1998). Cell wall elasticity and osmotic potentials (*Ψ_π_*) are closely linked to changes in cell volume (e.g. Δ*V*/Δ*Ψ* = *V*/(*ɛ* + *Ψ_π_*); Tyerman 1982), and it is possible that rigid tissues can limit dehydration as both low *Ψ_π_* and high *ɛ* together are effective means of maintaining vital cellular water content (Cheung *et al.* 1975; Bartlett *et al.* 2012). This concept, termed the ‘cell water conservation hypothesis’, likely plays an important role in marine and coastal plants that accumulate ions or other solutes as an osmoregulatory response to elevated environmental salts.

Although plants with comparatively rigid tissues tend to lose turgor faster than plants with more flexible tissues (given comparable decreases in cell volume), loss in turgor may promote other important water-stress responses including stomatal closure, wilting and leaf rolling (Barker *et al.* 1993). Changes in *ɛ* may be induced by developmental and/or environmental factors including plant or tissue age (Bowman and Roberts 1985; Moore and Cosgrove 1991; Merchant *et al.* 2007), hysteresis from water stress altering cell wall viscoelastic properties (Nolte and Schopfer 1997; Schopfer 2006), drought (Joly and Zaerr 1987; Clifford *et al.* 1998; Touchette *et al.* 2007) and salinity (Bolaños and Longstreth 1984; Nabil and Coudret 1995; Touchette *et al.* 2009*a*). Indeed, these factors often work in concert, wherein the specific response of *ɛ* to water stress may depend on, for example, the age of the tissue during drought (Saito and Terashima 2004). While salinity- and drought-strain are physiologically similar in plants (both fostering cellular dehydration, ion accumulation, formation of reactive oxygen species and diminished photosynthetic capacity), the ratio of accumulated ions will be markedly different due to selective ion uptake during salinity stress (Kirst 1989; Bartels and Sunkar 2005; Touchette 2007). Moreover, it would appear that salt stress promotes a disproportionally higher *ɛ* and lower *Ψ_π_* in coastal–marine plants in comparison to drought stress which fosters smaller increases or even decreases in tissue elasticity (Touchette *et al.* 2007, 2009*a*). In some plant tissues, higher salinities can promote up to a 30-fold increase in *ɛ* along with a concomitant accumulation of compatible osmolytes that lower *Ψ_π_* (Bolaños and Longstreth 1984). In contrast, one study found that three of five freshwater perennials receiving drought had decreased *ɛ* and/or increased *Ψ_π_* (Touchette *et al.* 2007).

As mentioned, several studies have shown strong associations between elevated environmental salinities and increased tissue rigidities (Bolaños and Longstreth 1984; Salpeter *et al.* 2012). These findings, in general, suggest that marine halophytes should have higher *ɛ* values compared with freshwater or terrestrial plant species. To our knowledge, however, no study has been conducted to fully consider this relationship in hydrophytic angiosperms. Therefore, the purpose of this investigation was to test the hypothesis that coastal or marine aquatic and wetland plants (exposed to natural and/or artificial salinities) will have higher *ɛ* relative to plants residing in freshwater systems. We also wanted to consider how *Ψ_π_* and symplastic water content (*θ*_sym_) interact with *ɛ* in promoting optimal water relations in plants from different aquatic systems. We intentionally focused on aquatic and wetland plants residing in relatively stable hydrologies as plants previously exposed to low soil moistures may have altered physiological properties (e.g. tissue elasticity and solute content) that would promote greater tolerances towards future soil-water deficits (Morgan 1984; Touchette *et al.* 2007). Therefore, as prevailing water conditions may be an important determinant for *ɛ*, we wanted to avoid using plants from natural systems with unknown water histories (e.g. terrestrial or upland environments) and perhaps prior drought exposure.

## Methods

### Plant selection and classification

This study combined laboratory and/or field evaluations on selected aquatic and wetland plants along with a meta-analysis of reported physiological values from other published studies. For the purposes of this investigation, a species is considered hydrophytic if its probability of occurring in an aquatic and/or wetland system is 67 % or greater (i.e. designated as either a facultative- or an obligate-wetland plant by the United States Fish and Wildlife Service; Reed 1988). Within this context, we focused on values obtained from pressure–volume curves on vascular plants from three habitat types: freshwater, coastal and marine (Table 1). Freshwater plants included aquatic species that were emergent and/or grew just above the water table (e.g. along stream- or lake-banks). Coastal plants included species that were living in principally freshwater systems, but were in locations where periodic salt exposure was likely. For this study, coastal plants were typically <0.5 km from seawater and could occasionally be exposed to environmental salts from storm tides and/or seawater aerosols (Touchette *et al.* 2009*b*). Marine plants were those that resided directly in marine or brackish waters and were known to have some degree of salt tolerance.

**Table 1.** List of plant species use in this assessment. Data include species, taxonomic family, habitat (freshwater, coastal or marine), growth form (forb-herb, graminoid or shrub), environmental conditions at the time of collection (including salinity for marine plants; in practical salinity units [psu]) and the reference or source of the data.

Species	Family	Habitat	Growth/habit	Environ. conditions/salinity	Source
*Acorus americanus*	Acoraceae	Freshwater	Forb-herb	Bioretention basin (flooded)	This study
*Acorus americanus*	Acoraceae	Freshwater	Forb-herb	Greenhouse (flooded)	Romanello *et al.* (2008)
*Arundinaria gigantean*	Poaceae	Coastal	Graminoid	Damp soils 100 m from seawater	This study
*Atriplex hortensis*	Chenopodiaceae	Freshwater	Forb-herb	Greenhouse	Kachout *et al.* (2011)
*Bidens* sp.	Asteraceae	Freshwater	Forb-herb	Greenhouse (saturated soils)	This study
*Borrichia frutescens*	Asteraceae	Marine	Shrub	Upper salt marsh/32 psu	This study
*Carex alata*	Cyperaceae	Freshwater	Graminoid	Greenhouse (flooded)	Touchette *et al.* (2007)
*Carex alata*	Cyperaceae	Freshwater	Graminoid	Greenhouse (saturated soils)	This study
*Cephalanthus occidentalis*	Rubiaceae	Freshwater	Shrub	Lacustrine wet shoreline	This study
*Eleocharis* sp.	Cyperaceae	Freshwater	Graminoid	Greenhouse (flooded)	This study
*Halodule wrightii*	Cymodoceaceae	Marine	Forb-herb	Seawater (submerged)/32 psu	Wilson *et al.* (2010)
*Halodule wrightii*	Cymodoceaceae	Marine	Forb-herb	Seawater (submerged)/32 psu	Touchette (2007)
*Halophila ovalis*	Hydrocharitaceae	Marine	Forb-herb	Seawater (submerged)/32 psu	Tyerman (1982)
*Heteranthera limosa*	Pontederiaceae	Freshwater	Forb-herb	Lotic wet shoreline	This study
*Hibiscus moscheutos*	Malvaceae	Freshwater	Shrub	Lacustrine wet shoreline	This study
*Juncus effusus*	Juncaceae	Freshwater	Graminoid	Greenhouse (flooded)	Touchette *et al.* (2007)
*Juncus roemerianus*	Juncaceae	Marine	Graminoid	Lower salt marsh/18 psu	Touchette (2006)
*Juncus roemerianus*	Juncaceae	Marine	Graminoid	Greenhouse (flooded)/30 psu	This study
*Juncus roemerianus*	Juncaceae	Marine	Graminoid	Greenhouse (flooded)/30 psu	Touchette *et al.* (2009*a*)
*Juncus roemerianus*	Juncaceae	Marine	Graminoid	Greenhouse (flooded)/0 psu	Touchette *et al.* (2009*a*)
*Juncus* sp.	Juncaceae	Freshwater	Graminoid	Freshwater (flooded)	This study
*Justicia americana*	Acanthaceae	Freshwater	Forb-herb	Lacustrine emergent	This study
*Justicia americana*	Acanthaceae	Freshwater	Forb-herb	Greenhouse (flooded)	Touchette *et al.* (2007)
*Microstegium vimineum*	Poaceae	Freshwater	Graminoid	Greenhouse (flooded)	Touchette and Romanello (2010)
*Peltandra virginica*	Araceae	Freshwater	Forb-herb	Greenhouse (flooded)	Touchette *et al.* (2007)
*Polygonum amphibium*	Polygonaceae	Freshwater	Forb-herb	Lotic emergent	This study
*Pontederia cordata*	Pontederiaceae	Freshwater	Forb-herb	Greenhouse (flooded)	This study
*Posidonia australis*	Posidoniaceae	Marine	Forb-herb	Seawater (submerged)/32 psu	Tyerman (1982)
*Rosa palustris*	Rosaceae	Freshwater	Shrub	Lacustrine wet shoreline	This study
*Sabal minor*	Arecaceae	Coastal	Shrub	Damp soils 200 m from seawater	This study
*Sabal minor*	Arecaceae	Coastal	Shrub	Coastal maritime swamp	Touchette *et al.* (2012)
*Saururus cernuus*	Saururaceae	Freshwater	Forb-herb	Lacustrine wet shoreline	This study
*Saururus cernuus*	Saururaceae	Freshwater	Forb-herb	Greenhouse (flooded)	Touchette *et al.* (2007)
*Schoenoplectus pungens*	Cyperaceae	Freshwater	Graminoid	Lacustrine emergent	This study
*Schoenoplectus robustus*	Cyperaceae	Marine	Graminoid	Upper salt marsh/15 psu	This study
*Schoenoplectus tabernaemontani*	Cyperaceae	Freshwater	Graminoid	Lacustrine emergent	This study
*Sorghastrum* sp.	Poaceae	Freshwater	Graminoid	Greenhouse (flooded)	This study
*Spartina alterniflora*	Poaceae	Marine	Graminoid	Greenhouse (flooded)/0 psu	Touchette *et al.* (2009*a*)
*Spartina alterniflora*	Poaceae	Marine	Graminoid	Lower salt marsh/15 psu	This study
*Spartina alterniflora*	Poaceae	Marine	Graminoid	Greenhouse (flooded)/30 psu	Touchette *et al.* (2009*a*)
*Spartina patens*	Poaceae	Marine	Graminoid	Greenhouse (wet soil)/0–45 psu	Salpeter *et al.* (2012)
*Spartina patens*	Poaceae	Marine	Graminoid	Lower salt marsh/32 psu	This study
*Suaeda calceoliformis*	Chenopodiaceae	Marine	Forb-herb	Greenhouse/30 psu	Youngman and Heckathorn (1992)
*Suaeda calceoliformis*	Chenopodiaceae	Marine	Forb-herb	Greenhouse/50 psu	Youngman and Heckathorn (1992)
*Syringodium filiforme*	Cymodoceaceae	Marine	Forb-herb	Seawater (submerged)/32 psu	Wilson *et al.* (2010)
*Thalassia testudinum*	Hydrocharitaceae	Marine	Forb-herb	Seawater (submerged)/32 psu	Wilson *et al.* (2010)
*Typha angustifolia*	Typhaceae	Coastal	Forb-herb	Flooded 250 m from seawater	This study
*Typha latifolia*	Typhaceae	Freshwater	Forb-herb	Lotic emergent	This study
*Typha latifolia*	Typhaceae	Coastal	Forb-herb	Flooded 100 m from seawater	This study
*Typha* sp.	Typhaceae	Freshwater	Forb-herb	Lotic emergent	This study
*Zostera capricorni*	Zosteraceae	Marine	Forb-herb	Seawater (submerged)/32 psu	Tyerman (1982)

Plants were selected either from natural populations where hydrology was expected to be fairly static (e.g. lake or perennial stream populations) or from greenhouse-maintained hydrophytes grown in flooded or soil-saturated containers (20-L microcosms as described in Touchette *et al.* 2010) for more than 12 months. For the most part, collected plants were restricted to Atlantic coastal regions of North America, including Maryland, North Carolina and Vermont. During the collection of coastal and marine plants, ambient environmental salinities were recorded in surface or soil pore-waters located near the plant. Pressure–volume measurements were restricted to young fully expanded leaves to minimize differences attributed to the tissue developmental stage.

To complement data obtained on our cultured/collected samples and to expand the number of species used in this evaluation, we also incorporated values from other studies reported in the literature. For the most part, the data used were from known aquatic species (emergent or submersed plants) that were either collected or maintained in water-saturated environments (Table 1).

### Pressure–volume curves

A Scholander pressure chamber (model no. 1000; PMS Instrument Co., Albany, OR, USA; Scholander *et al.* 1965) was used to determine leaf-water potentials (*Ψ*_leaf_) on 5–10 plants per species (samples collected from different individuals with the quantity depending on the abundance or rarity of the species being considered). Prior to conducting pressure–volume analyses, leaves were fully submerged in deionized water and allowed to reach full turgor in darkness. During analysis, water deficits were established by exposing the samples to transpirational water loss on a laboratory bench. This process was favoured by Turner *et al.* (1984) as it minimized possible disequilibria of *Ψ* between apoplastic and symplastic tissues.

Pressure–volume curves were constructed by plotting the reciprocal of *Ψ*_leaf_ against relative water content (*θ*). In this case, *θ* was determined as follows:

$$\theta = \displaystyle{{({W_{\rm f} -W_{\rm d} } )} \over {({W_{\rm t} -W_{\rm d} } )}}$$

where *W*_f_ is the fresh weight recorded at the time of the *Ψ*_leaf_ measurement, *W*_t_ is the initial turgid weight and *W*_d_ is the oven dry weight (65 °C, until constant weight). First-order regressions were performed on the linear portion of the curves, which are equivalent to tissue osmotic potentials (*Ψ*_*π*_). By extension, this line was used to determine osmotic potential at full saturation ($\Psi _\pi ^{{\rm sat}} $), osmotic potential at turgor loss point ($ {\it \Psi} _\pi ^{{\rm tlp}} $) and symplastic water content (*θ*_sym_). Following *Ψ_π_* correction, bulk elastic modulus (*ɛ*) was obtained from the initial part of the curve as described by the following equation:

$$\varepsilon = \displaystyle{{\hbox{d}{\it \Psi} _{\rm p} } \over {\hbox{d}\theta }}\theta _{{\rm sym}} $$

where changes in turgor potential (*Ψ*_p_) were compared against changes in *θ* and symplastic water content (*θ*_sym_).

### Data analysis

For comparing species physiological characteristics (both data collected from our laboratory and values obtained from the literature), we used the mean value reported for each species with the exception of three seagrasses (*Halophila ovalis*, *Posidonia australis* and *Zostera capricorni*). For those exceptions, the reported values were maxima and *ɛ* were stationary volumetric elastic moduli (*ɛ*_s_) and not the more commonly reported instantaneous volumetric elastic moduli (*ɛ*_i_). Note, for comparative purposes, *ɛ*_i_ values tend to be higher than *ɛ*_s_ when recorded on the same plant (Tyerman 1982). Regression analyses were performed using Pearson product moment to identify correlates among physiological traits. Species-specific data were then combined into habitat types (i.e. freshwater, coastal or marine) to derive an overall grand mean. Because of the small sample sizes for coastal and marine plants, unequal replication and frequent occurrence of unequal variances, we elected to compare values classified within each habitat using a non-parametric Kruskal–Wallis one-way analysis of variance (KW one-way ANOVA) followed by Dunn's multiple comparisons test for *post hoc* evaluations. To further consider relationships between habitat type and physiological characteristics, we employed principal components analysis (PCA) as a reduction tool for plant physiological traits (i.e. *ɛ*, *θ*_sym_, ${\it \Psi} _\pi ^{{\rm sat}} $ and ${\it \Psi} _\pi ^{{\rm tlp}} $) specific to each habitat. Initial observations also indicated that growth form (graminoid, forb and shrub) may explain some physiological traits. Therefore, grand mean values classified by plant form were also compared using KW one-way ANOVAs followed by Dunn's multiple comparisons test. All comparisons were considered significant at *α* = 0.05.

## Results

In all, we considered a total of 38 aquatic and wetland plants, most of which were emergent freshwater species (*n* = 22; Table 1). Because of the selective pressures of growing in saline environments, we had considerably fewer coastal wetland (*n* = 4) and marine (*n* = 12) plant species (Table 1). In addition, we included multiple measurements of the same species (when available) to help elucidate within-species variability. Most of the within-species values reported in this study (including values obtained from the literature) were fairly comparable. Values for *Juncus roemerianus*, however, were markedly different (*ɛ* ranging from 4.5 to 31.3 MPa). This discrepancy in *J. roemerianus* was most likely attributed to the prevailing environmental salinities, wherein higher salinities appeared to promote greater tissue rigidity. Pearson correlation analyses revealed significant inverse correlations between *ɛ* and *Ψ_π_* (at both saturation and turgor loss point; *P* < 0.001) and, as expected, a strong correlation between ${\it \Psi} _\pi ^{{\rm sat}} $ and ${\it \Psi} _\pi ^{{\rm tlp}} $ (*r* = 0.935; Table 2). No significant correlations were observed between *θ*_sym_ and the other physiological parameters.

**Table 2.** Pearson correlation matrix for plant–water relation parameters, bulk modulus of elasticity (*ɛ*), osmotic potential at full saturation (*Ψ*_π_^sat^), osmotic potential at turgor loss point (*Ψ*_π_^tlp^) and symplastic water content (*θ*_sym_). Data from all species (the number of species was between 30 and 34, depending on data availability) were used regardless of habitat designation. The matrix includes correlation coefficients (*r*), probability values (*P* values) and number of comparisons made (*n*).

Parameter	Correlation	${\bi \Psi} _{\bi \pi}^{{\bf sat}} $	${\bi \Psi}_{\bi \pi}^{{\bf tlp}} $	*θ*_sym_
*ɛ*	Coefficient (*r*)	−0.735	−0.458	0.165
	*P* value	<0.001	<0.001	0.360
	*n*	34	31	33
${\it \Psi} _\pi ^{{\rm sat}} $	Coefficient (*r*)	–	0.935	0.231
	*P* value	–	<0.001	0.210
	*n*	–	31	31
${\it \Psi} _\pi ^{{\rm tlp}} $	Coefficient (*r*)	–	–	0.231
	*P* value	–	–	0.219
	*n*	–	–	30

**Figure 1.**
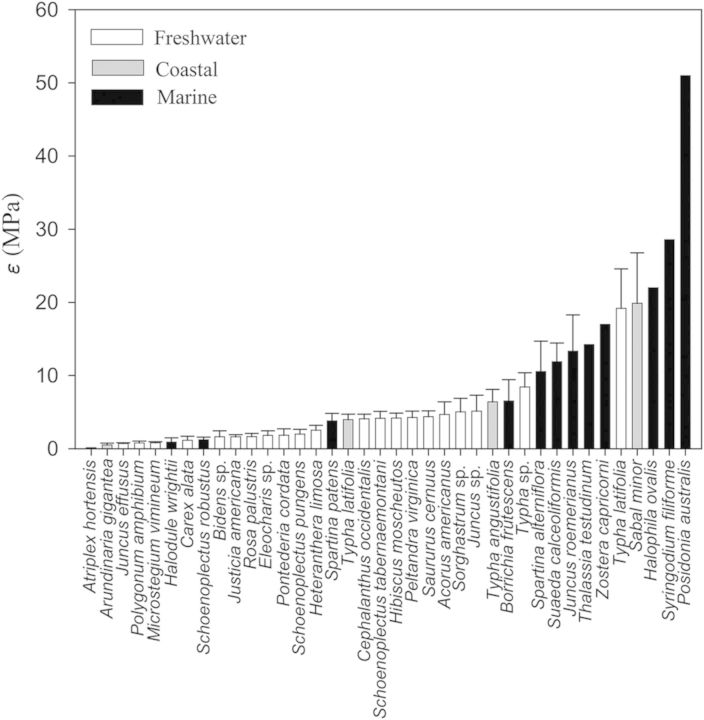
Bulk elastic moduli (*ɛ*) of aquatic and wetland plants considered in this study. Data include values reported for different species and their respective environmental conditions including freshwater (white bars), coastal (grey bars) and marine (black bars). Data, where applicable, are presented as means ± 1 standard error (SE).

**Figure 2.**
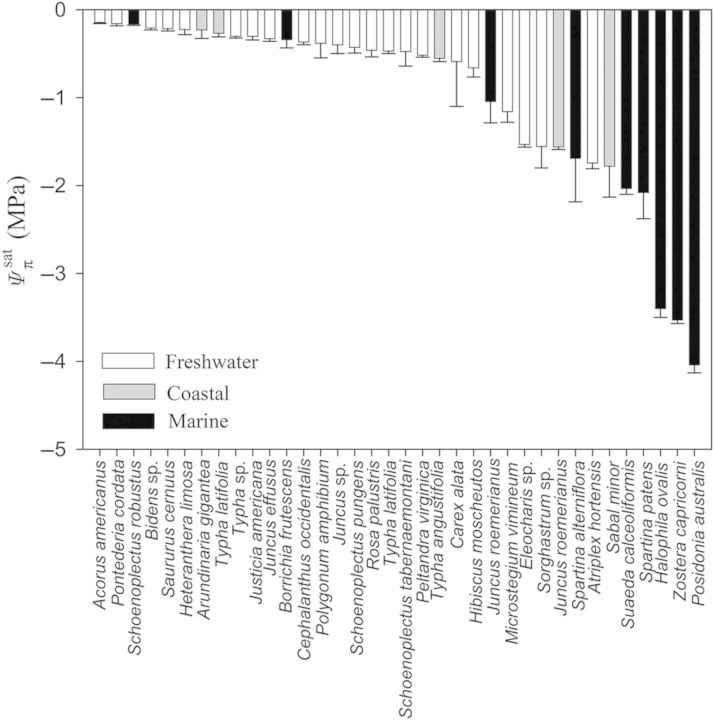
Solute potentials at full saturation (${\it \Psi} _\pi ^{{\rm sat}} $) of aquatic and wetland plants considered in this study. Data include values reported for different species and their respective environmental conditions including freshwater (white bars), coastal (grey bars) and marine (black bars). Data, where applicable, are presented as means ± 1 SE.

**Figure 3.**
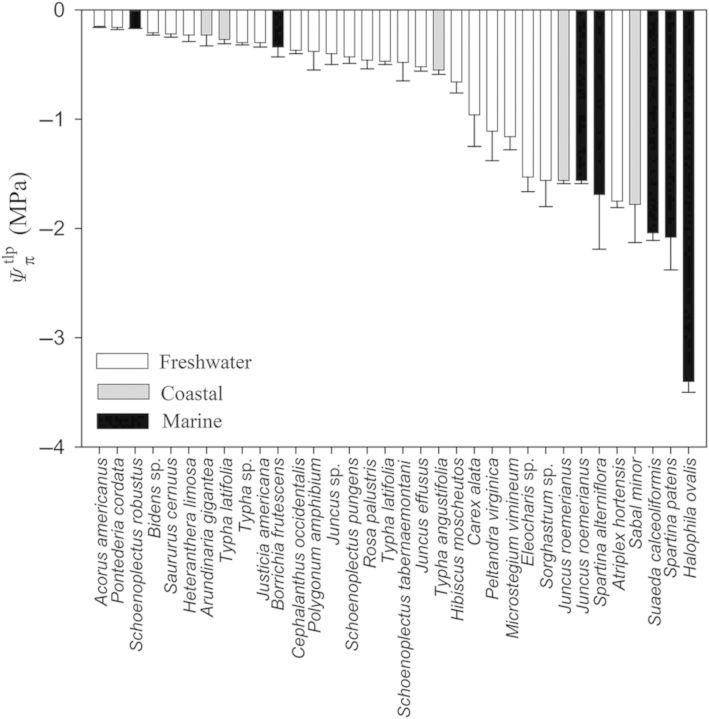
Solute potentials at turgor loss point (${\it \Psi} _\pi ^{{\rm tlp}} $) of aquatic and wetland plants considered in this study. Data include values reported for different species and their respective environmental conditions including freshwater (white bars), coastal (grey bars) and marine (black bars). Note that data were not available for a few marine plant species. Data, where applicable, are presented as means ± 1 SE.

When comparing across all species it was apparent that marine plants tend to have greater *ɛ* values (Fig. 1). That is, 8 of the 10 most rigid tissues were found in marine plants and only one freshwater plant (*Typha latifolia* living as a lotic emergent) had *ɛ* >10 MPa. Similarly, of the 25 species with the lowest tissue rigidity, 20 species were from freshwater systems. Nevertheless, there were three marine species with surprisingly elastic tissues, including the seagrass *Halodule wrightii* and the salt marsh emergents *Schoenoplectus robustus* and *Spartina patens* (Fig. 1). Unlike freshwater and marine plants, there were no notable trends in coastal plants that had *ɛ* values ranging from 0.5 ± 0.2 to 19.8 ± 6.9 MPa. As with tissue rigidity, there appeared to be habitat-specific responses with respect to ${\it \Psi} _\pi ^{{\rm sat}} $ and ${\it \Psi} _\pi ^{{\rm tlp}} $ (Figs 2 and 3). Marine plants tended to have lower ${\it \Psi} _\pi ^{{\rm sat}} $, wherein the lowest five observed values were marine species. Moreover, while most marine species had ${\it \Psi} _\pi ^{{\rm sat}} $ values lower than −1.0 MPa, most freshwater plants were well above −1.0 MPa (Fig. 2). As with ${\it \Psi} _\pi ^{{\rm sat}} $, reported values for ${\it \Psi} _\pi ^{{\rm tlp}} $ were also lower in marine plants (Fig. 3; but note fewer reported values as data were not available for some marine species).

**Figure 4.**
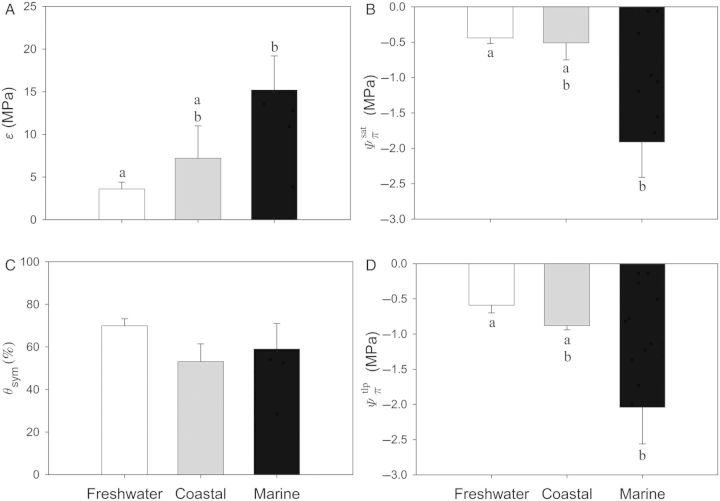
Bulk elastic moduli (*ɛ*; A), solute potentials at full saturation (${\it \Psi} _\pi ^{{\rm sat}} $; B), symplastic water content (*θ*_sym_; C) and solute potential at turgor loss point (${\it \Psi} _\pi ^{{\rm tlp}} $; D) for plants categorized as freshwater (white bars), coastal (grey bars) and marine (black bars). Statistical differences are indicated by the letters above the bars, wherein different letters identify significant differences among the three habitat types. Data are presented as means (grand means for each category as described in the Methods section) ± 1 SE.

**Figure 5.**
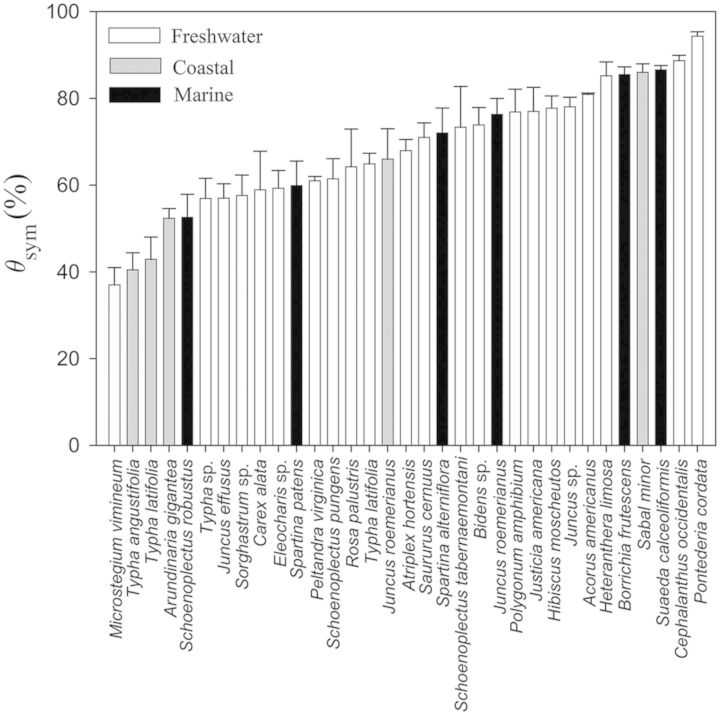
Symplastic water content (*θ*_sym_) of aquatic and wetland plants considered in this study. Data include values reported for different species and their respective environmental conditions including freshwater (white bars), coastal (grey bars) and marine (black bars). Note that data were not available for a few marine plant species. Data, where applicable, are presented as means ± 1 SE.

Statistical comparisons of plants residing in different habitats revealed significant differences between freshwater and marine plants (Fig. 4). In general, marine plants were 4.2 times more rigid than freshwater species (*P* < 0.001; Fig. 4A). Mean rigidity in coastal plants, although highly variable, was between freshwater and marine macrophytes and not significantly different from either group (*P* > 0.05). Solute potential was also different between marine and freshwater species (*P* < 0.001; Fig. 4), wherein ${\it \Psi} _\pi ^{{\rm sat}} $, for example, was 4.3 times lower in marine plants relative to freshwater emergent species. Moreover, while *Ψ_π_* in coastal plants were more similar to freshwater species, they were not significantly different from either freshwater or marine plants, likely attributed to high variability within this group (Fig. 4). Symplastic water content (*θ*_sym_), which ranged between 53.1 ± 8.3 and 70.0 ± 3.3 %, was not significantly different among plants from different habitat types (*P* = 0.073; Figs 4C and 5).

**Figure 6.**
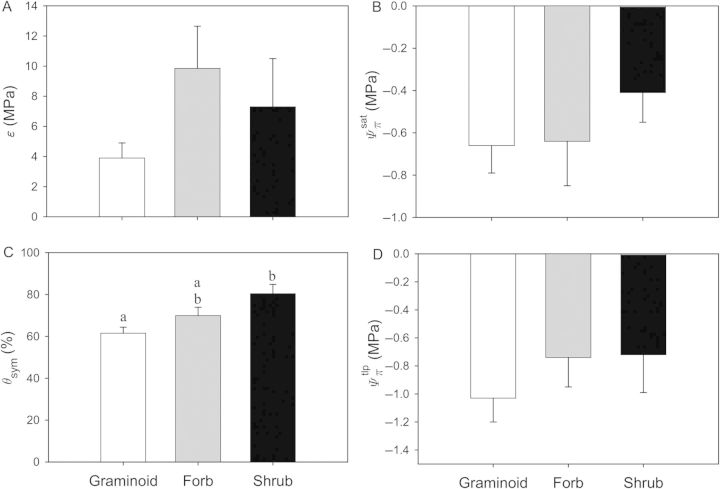
Bulk elastic moduli (*ɛ*; A), solute potentials at full saturation (${\it \Psi} _\pi ^{{\rm sat}} $; B), symplastic water content (*θ*_sym_; C) and solute potential at turgor loss point (${\it \Psi} _\pi ^{{\rm tlp}} $; D) for plants categorized by form including graminoids (white bars), forbs (grey bars) and shrubs (black bars). Statistical differences are indicated by the letters above the bars, wherein different letters identify significant differences among the three habitat types. Data are presented as means (grand means for each category as described in the Methods section) ± 1 SE.

While *θ*_sym_ appeared to be similar among plants residing in the three habitats explored in this study, there was a significant difference between *θ*_sym_ and plant growth form (irrespective of habitat type; Fig. 6C; *P* = 0.017). In this case, graminoids had significantly lower *θ*_sym_ than shrubs (*P* < 0.01). In contrast, there were no significant differences between growth form and *ɛ*, ${\it \Psi} _\pi ^{{\rm sat}} $ and ${\it \Psi} _\pi ^{{\rm tlp}} $ (*P* = 0.310, 0.597 and 0.221, respectively; Fig. 6).

**Figure 7.**
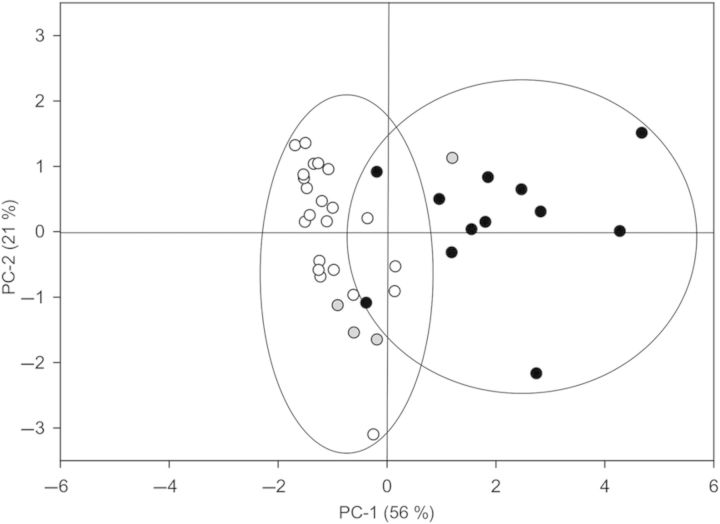
Principal components analysis of physiological traits (*ɛ*, *θ*_sym_, ${\it \Psi} _\pi ^{{\rm sat}} $ and ${\it \Psi} _\pi ^{{\rm tlp}} $) measured in different aquatic and wetland plant species including freshwater (white circles), coastal (grey circles) and marine (black circles). Percentages of explained variances for the first and second principal components (PC) are in parentheses. Note the pronounced horizontal tendencies between freshwater and marine species.

Principal components analysis comparing habitat and physiological responses among all 38 species agreed with the previous distinctions between marine and freshwater species (Fig. 7). Approximately 56 % of the variability in the data was accounted for in the first principal component. The cumulative variance was increased to 77 % when combined with the second component. Based on eigenvector loadings (Table 3), the first principal component was mostly associated with *ɛ*, ${\it \Psi} _\pi ^{{\rm sat}} $ and ${\it \Psi} _\pi ^{{\rm tlp}} $, and these three parameters contributed the most towards distinguishing between marine and freshwater plants (as habitat-group distinctions elicited strong horizontal tendencies; Fig. 7). The second principal component was overwhelmingly associated with *θ*_sym_ (with an eigenvector of 0.92) and was unable to provide further resolution among the three plant groups (i.e. lacking strong vertical tendencies; Fig. 7). As with other physiological characteristics, coastal plants were clustered between marine and freshwater plants and were indistinguishable from other plant habitat types.

**Table 3.** Matrix of eigenvector loadings for physiological predictors for freshwater, coastal and marine plants. Most of the variance was explained by the first four principal components (97.1 %; 77.3 % was explained by the first two principal components).

Variable	Principal component			
	1	2	3	4
Habitat	0.4854	−0.0142	−0.0723	0.8694
*ɛ*	0.4588	0.3445	−0.6181	−0.3294
*θ*_sym_	−0.0938	0.9201	0.3652	0.1001
${\it \Psi} _\pi ^{{\rm sat}} $	−0.5604	−0.0399	−0.0368	0.2584
${\it \Psi} _\pi ^{{\rm tlp}} $	−0.4806	0.1815	−0.6914	0.2427

## Discussion

In response to sudden changes in plant-water status (e.g. water availability and/or environmental salinity), turgor pressure in tissues may increase or decrease as water moves in the direction of the osmotic gradient. The degree of water flux is largely dependent on hydraulic conductivity of cell membranes, intracellular osmotic pressure, cell volume and elastic properties of cell walls (Tyerman 1982; Kramer and Boyer 1995; Touchette 2007). Cell wall elasticity can vary greatly among plant species, with reported ranges of *ɛ* from 0.06 MPa in *Halicystis parvula* to 70 MPa in *Chara corrallina* (Dainty *et al.* 1974; Graves and Gutknecht 1976). In some instances, flexible cell walls (low *ɛ*) can benefit plants inhabiting systems with dynamic salinity fluctuations, as cell walls expand or contract in order to maintain osmotic equilibrium with the environment (Kirst 1989; Touchette 2006). In hyperosmotic conditions, for example, lower *ɛ* can foster positive turgor pressures during periods of water efflux. These physiological processes are sometimes employed in estuarine macroalgae, where changes in osmolyte levels (i.e. *Ψ_π_*) and cell volume are important mechanisms for osmotic adjustment (Kirst 1989). For these algae, sudden changes in water salinity may be balanced by expanding or shrinking of comparatively thin cell walls.

Based on observations from this study, however, only a few marine angiosperms actually have relatively flexible cell walls (*ɛ* < 10 MPa). This response may be explained, in part, by the saline dynamics within the particular system. *Schoenoplectus robustus*, for example, had the eighth lowest *ɛ* of the 38 species considered. Although classified as a salt marsh plant, *S. robustus* resides along the mid-to-upper boundaries of salt marsh systems where salinities tend to be relatively low (sometimes freshwater) with sporadic inundations of higher saline waters (Ustin *et al.* 1982; Crain *et al.* 2004). Indeed, Ustin *et al.* (1982) were able to demonstrate that among the different salt marsh zones, the regions dominated by *S. robustus* maintained the greatest seasonal fluctuations in salinity. Not only would having comparatively flexible tissues benefit *S. robustus* in this osmotically dynamic environment, but apparently *S. robustus* can lie dormant as tubers for up to 2 years to avoid extended periods of hypersalinity (Ustin *et al.* 1982; Crain *et al.* 2004). Furthermore, *S. robustus* had comparatively high *Ψ_π_* (the third highest among all species in this study); thus any substantial decline in cell volume may not confine existing solutes to levels where metabolic functions are compromised (e.g. salting out metabolites). *Spartina patens*, which also has relatively flexible tissues, is common along the upper reaches of salt marshes where it is fed by shallow freshwater systems. Unlike *S. robustus*, *S. patens* can have comparatively low *Ψ_π_* (−2.14 MPa), although highly variable with some reported potentials as high as −0.25 MPa. Apparently, *S. patens* can undergo extended periods of salt avoidance when exposed to hypersaline conditions, and perhaps these delays in modifying plant–water relations may contribute to a greater range in both *ɛ* and *Ψ_π_* (Salpeter *et al.* 2012). That is, *S. patens* will likely maintain low *ɛ* and high *Ψ_π_* when exposed to short periods of high salinity, but longer exposures (>5 weeks) may foster physiological alterations that include higher *ɛ* and lower *Ψ_π_* (Salpeter *et al.* 2012). The seagrass *H. wrightii* had the sixth most flexible tissue of the 38 species considered and, as with the other 2 species, resides in osmotically dynamic systems (Touchette 2006). That is, *H. wrightii* has been shown to thrive in the Guadalupe estuary where salinities can fluctuate from 5 to 25 practical salinity units (psu) over a few months, with periodic spikes in salinities up to 55 (Dunton 1996). We argue that for *S. robustus*, *S. patens* and *H. wrightii* to survive in these highly dynamic euryhaline environments, like estuarine macroalgae, maintaining flexible cell walls could be advantageous provided cytoplasmic ion content remains low and/or *Ψ_π_* remains high (including osmotic adjustments attributed to compatible solutes and/or vacuolar sequestration of ions; Kirst 1989; Touchette 2007).

While a few marine plants have relatively flexible cell walls, most studies indicate that plants residing in saline environments will have rigid tissues (Ike and Thurtell 1981; Melkonian *et al.* 1982; Bolaños and Longstreth 1984). The results from this study, with the aforementioned exceptions, support this previously untested assertion. Here 9 of the 10 species with the highest *ɛ* values were either marine or coastal plants. For plants with comparatively high *ɛ*, a corresponding reduction in osmotic potentials could help prevent dehydration and shrinkage during periods of lower *Ψ*_soil_ (Cheung *et al.* 1975; Bartlett *et al.* 2012). Rigid cells may also be advantageous in marine plants that tend to sequester ions for osmotic balance, as diminishing cell volume would confine existing solutes and ions to a level that could disrupt cellular processes (Touchette 2007). This notion is supported by the tendency for plants with high *ɛ* to also have low *Ψ_π_* (suggesting high cellular solute content). Therefore, plants residing in more stable marine environments (including lower salt marsh areas or open coastal waters) would benefit from having thicker cell walls with relatively low elasticity (Kirst 1989; Touchette 2007; Bartlett *et al.* 2012).

In contrast to marine species, freshwater plants generally have lower *ɛ* and higher *Ψ_π_*. Although we intentionally focused on plants from areas with more stable hydrologies, freshwater wetlands are transitional areas between terrestrial and deep-water habitats and therefore many of the resident species are physiologically adapted to periodic water deprivation from water-table drawdown and/or drought (Cowardin *et al.* 1979; Wilcox *et al.* 2002). For these plants, a small *ɛ* can facilitate turgor maintenance in cells as tissue water declines (Kozlowski *et al.* 1990). Indeed water stress may promote greater turgor in some plants following water repletion (Kikuta and Richter 1986; Saito and Terashima 2004). Maintaining turgor and avoiding plasmolysis are important in plants as high turgor pressure has been shown to enhance membrane transport, plant defence responses, cytoskeleton stability and cytoplasmic streaming (Mellersh and Heath 2001; Hayashi and Takagi 2003; Heidecker *et al.* 2003). Therefore, with higher *Ψ_π_* limiting the likelihood of confining solutes to the point of metabolic disruption (as in marine species), freshwater species tend to maintain flexible cells that minimize the likelihood of zero turgor or plasmolysis.

Finally, although there were no significant differences in *θ*_sym_ among the three habitats considered in this study, we did observe that growth form may be an important component in determining the level of *θ*_sym_ in wetland plants. In this case, graminoids had significantly lower *θ*_sym_ than shrubs. One possible explanation for this difference, as well as the overall low *θ*_sym_ reported in aquatic grasses and forbs, is the predominance of aerenchyma that often develops in grasses and forbs in response to reduced anaerobic substratums (Jackson and Armstrong 1999). In this case, the substantial increase in apoplastic volume (due to aerenchyma development) within tissues could act, in part, as reservoirs for maintaining comparatively higher apoplastic water and consequently a proportionally lower symplastic water content (Gribble *et al.* 1998). The use of aerenchyma as a possible apoplastic water reservoir, along with other roles (e.g. a lacunar system that provides oxygen to roots residing in hypoxic/anoxic sediments; Grosse and Mevi-Schütz 1987), could benefit emergent wetland plants by providing water to the cells during periods of soil-water deficit or drought.

## Conclusions

In this study, we were able to demonstrate that marine plants, by and large, do have higher *ɛ* and lower *Ψ_π_*. By maintaining rigid tissues and lower *Ψ_π_*, marine plants may be able to both prevent dangerous dehydration and achieve tolerance of lower *Ψ*_soil_ (i.e. the ‘cell water conservation hypothesis’). Maintaining higher cellular water content could help minimize metabolic disruptions that may develop when existing solutes, already in high concentrations, are confined into smaller spaces during water efflux. In contrast, freshwater plants tend to have lower *ɛ* and higher *Ψ_π_*. Flexible tissues would allow for appreciable water loss while maintaining positive turgor pressure. In this case, because cellular solute content is low (i.e. high *Ψ_π_*) there may be greater probability that physiological disruptions will occur due to plasmolysis rather than to solute confinement.

## Sources of Funding

This study was supported by the North Carolina Sea Grant (NCSG), UNC Water Resources Research Institute (WRRI) and the Japheth E. Rawls Foundation.

## Contributions by the Authors

Experiments were carried out by all three authors. Compilation of data from previous published studies was conducted by B.W.T. and S.E.M. Data and statistical analyses were performed by all authors. The manuscript was written by B.W.T. and S.E.M.

## Conflicts of Interest Statement

None declared.
